# Variability in Prebiotic Carbohydrates in Different Market Classes of Chickpea, Common Bean, and Lentil Collected From the American Local Market

**DOI:** 10.3389/fnut.2019.00038

**Published:** 2019-04-03

**Authors:** Niroshan Siva, Pushparajah Thavarajah, Shiv Kumar, Dil Thavarajah

**Affiliations:** ^1^Plant and Environmental Sciences, Poole Agricultural Center, Clemson University, Clemson, SC, United States; ^2^Biodiversity and Integrated Gene Management Program, International Centre for Agricultural Research in the Dry Areas (ICARDA), Rabat-Institute, Rabat, Morocco

**Keywords:** pulse crops, low digestible carbohydrates, prebiotic carbohydrates, resistant starch, amylose

## Abstract

Pulse crops such as lentil, common bean, and chickpea are rich in protein, low digestible carbohydrates, and range of micronutrients. The detailed information of low digestible carbohydrates also known as “prebiotic carbohydrate” profiles of commonly consumed pulse market classes and their impact on human health are yet to be studied. The objective of this study was to determine the profiles of prebiotic carbohydrates in two commonly consumed lentil market classes, seven common bean market classes, and two chickpea market classes. After removing fat and protein, total carbohydrates averaged 51/100 g for lentil, 53/100 g for common bean, and 54/100 g for chickpea. Among the portion of total carbohydrates, lentil showed 12/100 g of prebiotic carbohydrates (sugar alcohols, raffinose family oligosaccharides, fructooligosaccharides, hemicellulose, cellulose, and resistant starch), 15/100 g in common bean, and 12/100 g in chickpea. Prebiotic carbohydrate concentrations within the market classes for each crop were significantly different (*P* < 0.05). In conclusion, these three pulses are rich in prebiotic carbohydrates, and considering the variation in these concentrations in the present materials, it is possible to breed appropriate market classes of pulses with high levels of prebiotic carbohydrates.

## Introduction

Carbohydrates are widely present in plants and animals and are used as an energy source to fulfill metabolic requirements (1). Carbohydrates are classified into three major groups, simple sugars, oligosaccharides, and polysaccharides or complex carbohydrates, based on their chemical structure. Complex carbohydrates have a degree of polymerization 10 or more than the simple and oligosaccharides. Prebiotic carbohydrates, a category of complex carbohydrates also known as low digestible carbohydrates, are defined as “*a selectively fermented ingredient that allows specific changes, both in the composition and/or activity in the gastrointestinal microflora that confers benefits upon host well-being and health*” (2, 3). A prebiotic carbohydrate is a specific colonic nutrient that acts as a biosynthetic precursor for human microbiota activity. Classification of a food as a prebiotic carbohydrate requires that the ingredient: (1) resists digestive processes in the upper part of the gastrointestinal tract, (2) is fermented by intestinal microbiota, and (3) selectively stimulates growth and activity of health-promoting bacteria (2). Simple carbohydrates are comprised of one sugar unit (monosaccharides) or two sugar units (disaccharides) that are easily digestible, whereas oligosaccharides have 3 to 10 sugar units and complex carbohydrates feature more than ten sugar units (polysaccharides) (4).

Complex carbohydrates provide prebiotic health benefits by modulating healthy gut bacteria (5). Whole grains are rich in prebiotic carbohydrates, but most food processing techniques remove prebiotic carbohydrates, especially in cereals, i.e., white bread and breakfast cereal, so consumption of such foods can lead to an increased risk of obesity and related non-communicable diseases (6). Pulse crops, such as lentil (*Lens culinaris* Medikus.), common bean (*Phaseolus vulgaris* L.), and chickpea (*Cicer arietinum* L.) are consumed as whole foods and require minimal or no processing, and therefore contain higher amounts of prebiotic carbohydrates than processed cereals and other grains (7–9). Diets rich in prebiotic carbohydrates change the gut microbial composition, lead to production of fatty acids (acetate, butyrate, and propionate), regulate intestinal movement, and prevent constipation (2). Additionally, such diets tend to increase mineral absorption and reduce obesity risk by regulating blood glucose and cholesterol levels (10). However, the current daily intake of prebiotic carbohydrates in Western populations is <50% of the recommended daily allowance (RDA) (11), but can be increased by incorporating pulses in the diet.

The benefits of prebiotic carbohydrates are not limited to humans, but also extend to plant health by increasing stress tolerance to cold and drought. For example, leaf raffinose family oligosaccharides (RFOs) enhance drought (12), chilling (13, 14), and freezing tolerance in plants (15). Further, sugar alcohols (SAs; sorbitol and mannitol) increase tolerance to chilling (16), drought (17), and salinity (18, 19). RFOs and SAs act as osmolytes to maintain cell structure during drought and salt stress (12, 20) and as antioxidants to neutralize the reactive oxygen species that cause cell damage (13, 14, 21). Further, SAs and RFOs act as signaling compounds for biotic stress caused by insects and pathogens (22, 23).

Current annual lentil, common bean, and chickpea production around the world is ~6, 12, and 26 million tons, respectively (24). With climate change, future pulse crop production might be limited because of increased drought and temperatures. As such, developing climate resilient and nutritionally superior cultivars via plant breeding and selection is essential for future pulse crop improvement and global food security (25). A 100 g serving of lentil contains 1–2 g of SA, 5–6 g of RFO, 0–1 g of fructooligosaccharides (FOS), and 2–8 g resistant starch (RS) (9). However, very limited information in terms of detailed profiles of prebiotic carbohydrates is available for other pulses, including chickpea and common bean. The objective of this study was to identify and quantify prebiotic carbohydrate profiles (simple sugars, SA, RFO, FOS, RS, cellulose, hemicellulose, amylose) in two lentil market classes (red and green), seven common bean market classes (small red, cranberry, great northern, light red kidney, black, navy, and pinto), and two chickpea market classes (desi and kabuli).

## Materials and Methods

### Materials

Chemicals used for high performance anion exchange chromatography (HPAE) and enzymatic assays were purchased from Fisher Scientific (Asheville, NC, USA), Sigma-Aldrich (St. Louis, MO, USA), and VWR International (Satellite Blvd, Suwanee, GA, USA). Distilled and deionized water (ddH_2_O) with a resistance of ≥18.2 MΩ (NANO-pure Diamond, Barnstead, IA, USA) was used in these analyses.

### Lentil, Common Bean, and Chickpea Seeds

Approximately 4 kg of five commercially available lentil seed samples from two market classes (red and green) were collected from the Northern Pulse Growers Association, ND, USA. The red market class included whole seed (with seed coat), dehulled (whole seed without seed coat), and dehulled split (split seed without seed coat) and the green market class included whole seed and dehulled split (Table 1). Samples (~2 kg) of seven commercially available common bean market classes (small red, cranberry, great northern, light red kidney, black, navy, and pinto) grown in the USA were obtained from local grocery stores, and two chickpea market classes (desi and kabuli) were obtained from a commercial pulse distributor (AGT Foods, Bismarck, ND, USA) (Table 1). These different pulse seed sample were collected from regional pulse distributors and local market, therefore additional information on growing conditions, soil management, and variety information were not available.

**Table 1:** Description of pulse market classes used in this experiment.

**Type**	**Market class**	**Commercial form**	**1,000 seed weight (g)**
Lentil	Red	Whole (with seed coat)	29
		Dehulled dehulled	33
		Dehulled split	16
	Green	Whole (with seed coat)	46
		Dehulled split	45
Common bean	Small red	Whole (with seed coat)	315
	Cranberry	Whole (with seed coat)	569
	Great northern	Whole (with seed coat)	338
	Light red kidney	Whole (with seed coat)	593
	Black	Whole (with seed coat)	182
	Navy	Whole (with seed coat)	198
	Pinto	Whole (with seed coat)	344
Chickpea	Desi	Whole (with seed coat)	228
	Kabuli	Whole (with seed coat)	473

Samples were cleaned by hand, homogenized, subsampled, and ground to a 1-mm particle size using a cyclone mill (CT 193 Cyclotec Sample Mill, FOSS North America, MN, USA). The treatment design was a completely randomized design with five lentil types, seven common bean types, and two chickpea types (*n* = 14) and three replicates (*n* = 3), for a total of 42 (*n* = 42).

### Fat and Protein Removal

Ground seed samples were dried at 100–102°C for 3 h. Fat was removed with hexane at 90°C for 2 h in an ANKOM extractor (XT15, Macedon, NY, USA). Defatted samples were treated with 0.2% NaOH (1:6; w/v) in a water bath at 45°C for 90 min to remove protein (26, 27). Samples were then blended for 2 min and centrifuged at 3,000 x g (Fisher Scientific, Waltham, MA, USA) for 15 min. The supernatant was discarded, and the top layer was removed. Ten mL of ddH_2_O were added, the solution was mixed and centrifuged, and the supernatant and top layer was removed. This process was repeated until the top yellow layer no longer visible. The suspension was re-suspended with 10 mL of ddH_2_O and adjusted to a pH of ~7 with 50 mM HCl (27). Following centrifugation, samples were washed three times with ddH_2_O and air dried at 60°C overnight.

### Low Molecular Weight Carbohydrates (LMWC)

Ground seed samples (500 mg) were weighed into 15-mL polypropylene conical tubes. Ten mL of ddH_2_O were then added to the tubes, which were incubated for 1 h at 80°C as per Muir et al. (28). Samples were centrifuged at 3,000 x g for 10 min. An aliquot (1 mL) of the supernatant was diluted with 9 mL of ddH_2_O, and the diluted supernatant was filtered through a 13 mm × 0.45 μm nylon syringe filter (Fisher Scientific, Waltham, MA, USA) prior to HPAE analysis.

Low molecular weight carbohydrate concentrations (SA, RFO, and FOS) were measured using HPAE (Dionex, ICS-5000, Sunnyvale, CA, USA) according to a previously published method (29). SA, RFO, and FOS were determined by running the mobile phases (A: 100 mM sodium hydroxide/600 mM sodium acetate; B: 200 mM sodium hydroxide; C: ddH_2_O) at a flow rate of 1 mL/min through a CarboPac PA1 column [250 × 4 mm; Dionex, CA, USA] connected to a CarboPac PA1 guard column (50 × 4 mm; Dionex, CA, USA). The total run time was 25 min. Detection was carried out using a pulsed amperometric detector (PAD; ICS-5000, Thermo Scientific, Waltham, MA, USA) with a working gold electrode and a silver-silver chloride reference electrode at 2.0 μA. Sugar alcohols (sorbitol and mannitol), RFO (raffinose, stachyose, and verbascose), and FOS (kestose and nystose) were identified and quantified using pure standards (>99%), and low molecular weight carbohydrate concentrations were detected within a linear range of 3–1,000 μg/g with a minimum detection limit of 0.2 μg/g. A lab reference (CDC Redberry lentil) was used to ensure the accuracy and reproducibility of detection. The peak areas of the external reference, glucose (100 ppm), SA (3–1,000 ppm), RFO (3–1,000 ppm), and FOS (3–1,000 ppm) were routinely analyzed for method consistency and detector sensitivity, with an error of <5% (9). The concentration of LMWC in the samples (C_s_) was calculated according to C_s_ = (C_f_ × V) / m, where C_f_ is the filtrate concentration obtained from HPAE, V is the final diluted volume, and m is the mass of the sample (moisture corrected). Unidentified compound concentrations were determined based on of those identified carbohydrate peak areas that were very closest to retention times.

### Hemicellulose

Samples weighing 500 mg were loaded into 15-mL polypropylene conical tubes, which were incubated with 5 mL of 7% (w/w) HCl at 55°C for 120 min followed by centrifugation at 3,000 x g for 10 min (30). Concentrations of arabinose and xylose were measured using the HPAE-PAD method described above. Hemicellulose concentration was reported as the summation of arabinose and xylose concentrations, and then multiplied by 0.9. Pectin concentration was not measured.

### Cellulose

Cellulose was measured using enzymatic hydrolysis of cellulose (31). Cellulase enzyme (extracted from *Aspergillus niger*, 1 U of enzyme liberates 1.0 μmole of glucose at 37°C for 1 h incubation) was purchased from Sigma-Aldrich, St. Louis, MO, USA. Samples (100 mg) were weighed into 15-mL polypropylene conical tubes. An aliquot (3.5 mL) of cellulase (34 U/mL in 50 mM citrate buffer, pH 4.7) was added and the mixture incubated in a water bath (Orbit shaker bath, Lab Line Instruments Inc., Melrose Park, ILL) with a rotary shaker (200 rpm) at 37°C for 10 h (31). Tubes were then centrifuged at 3,000 x *g* for 10 min and 1 mL of the supernatant then diluted with 19 mL of ddH_2_O. The total glucose concentration resulting from cellulose hydrolyzation was measured using an enzymatic assay (32). Aliquots (0.1 mL) of diluted solution and glucose standard (1 mg/mL) were added separately to 10-mL round bottom glass tubes. Then, 3 mL of GOPOD reagent (12,000 U/L glucose oxidase, 650 U/L peroxidase, and 0.4 mM 4-aminoantipyrine, pH 7.4) were added to each tube, which were then incubated in a water bath at 50°C for 20 min. The absorption of the samples was measured using a spectrophotometer (Genesys 20, Thermo Scientific, NC, USA) at 510 nm (the absorbance value of the glucose standard) to determine the concentration of glucose in the samples. The cellulose concentration was determined by multiplying the glucose concentration by 0.9 (the ratio of free glucose to anhydro-glucose that occurs in cellulose).

### Resistant Starch

RS concentrations were determined according to McCleary and Monaghan (33) and Megazyme (32). Ground samples (500 mg) were incubated with 4 mL of 100 mM sodium malate (pH 6) containing α-amylase (10 mg/mL) and amyloglucosidase (3 U/mL) for 16 h in a water bath (37 °C) with 200 strokes/min vertical shaking (Orbit shaker bath, Lab Line Instruments Inc., Melrose Park, IL, USA). After incubation, 4 mL of 95% ethanol were added, and the samples were then centrifuged at 1,500 x g for 10 min at room temperature. The pellets were re-suspended with 6 mL of ethanol (50% v/v), centrifuged, and decanted. The resuspension and centrifugation process were done two times. Supernatants from the three centrifugations were pooled and brought to a volume of 100 mL with ddH_2_O. The pellets were dissolved in 2 mL of potassium hydroxide (2 M) in an ice bath (~0°C) while stirring with a magnetic stirrer for 20 min. The suspensions were diluted with 8 mL of sodium acetate buffer (1.2 M, pH 3.8), with 0.1 mL of 3,300 U/mL amyloglucosidase then immediately added followed by incubation at 50°C for 30 min. The suspension was then centrifuged at 1,500 x g for 10 min at room temperature. Aliquots (0.1 ml) of both the supernatant containing the RS fractions and the diluted washings containing the soluble starch (SS) fractions were transferred separately to 10-mL glass tubes. A reagent blank was prepared using 0.1 mL sodium acetate buffer (pH 4.5). An aliquot (3 mL) of GOPOD reagent was added to each tube, which were incubated in a water bath at 50°C for 20 min. Absorption was measured using a spectrophotometer (Genesys 20, Thermo Scientific, NC, USA) at 510 nm. Starch fractions were calculated as follows:

$$\begin{array}{l} \text{RS=}\frac{\text{X }\times \;({\text{Abs}}_{\text{sample}})}{\left({\text{Abs}}_{\text{glucose}}\times \;{\text{W}}_{\text{sample}}\right)},\\ \text{SS=}\frac{\text{Y }\times \;({\text{Abs}}_{\text{sample}})}{\left({\text{Abs}}_{\text{glucose}}\times \;{\text{W}}_{\text{sample}}\right)}, \end{array}$$

where Abs_sample_ and Abs_glucose_ are the absorbance value of sample and glucose corrected against reagent blank, respectively; W_sample_ is the moisture corrected weight of sample; and X and Y are the dilutions factors for RS and SS, respectively. Regular corn starch [RS concentration 1.0 ± 0.1% (w/w)] was used to verify the data, and batches were checked regularly to ensure an analytical error of <10%.

### Amylose and Amylopectin

Amylose levels were determined using an enzymatic assay (34, 35). Samples (20–25 mg) of defatted and deproteinated flour were transferred to 15-mL screw capped polypropylene conical tubes. An aliquot (1 mL) of dimethyl sulphoxide (DMSO; 99.5% v/v) was added to each tube, which were heated for 1 min in a boiling water bath. The tube contents were then vigorously mixed in a high-speed vortex and heated for 15 min in a boiling water bath. The tubes were cooled to room temperature, and an aliquot (2 mL) of ethanol (95% v/v) added during continuous stirring. Then 4 mL of ethanol were added to the samples, which were allowed to stand for 15 min after thorough mixing. The tubes were centrifuged at 2,000 x g for 5 min, and the supernatant discarded. Two mL of DMSO were added, and the samples heated for 15 min in a boiling water bath with occasional mixing. Immediately after their removal, 4 mL of concanavalin A (Con A) buffer (180 mM sodium acetate buffer, pH 6.4) were added to the samples, which were mixed thoroughly. The contents were diluted with Con A buffer to 25 mL (Solvent A).

Aliquots (1 mL) of diluted solvent A were transferred to 2-mL microfuge tubes to which 0.5 mL of lectin Con A solution (6 mg/mL) was added. The tubes were mixed gently by repeated inversion and incubated for 1 h at room temperature followed by centrifugation at 14,000 x g for 10 min. The supernatant (1 mL) was transferred to a 15-mL centrifuge tube and 3 mL of sodium acetate buffer (100 mM, pH 4.5) then added. The contents were mixed in a boiling water bath for 5 min and incubated at 40°C for 5 min. Four mL of 100 mM sodium acetate buffer were added to 0.5 mL of solvent A. An aliquot (0.1 mL) of amyloglucosidase (333 U/ml)/ α-amylase enzyme (67 U/mL) was added to the tubes containing either diluted solvent A or con A supernatant, which were then incubated at 40°C for 10 min followed by centrifugation at 2,000 x g for 5 min. An aliquot (4 mL) of GOPOD reagent was added to 1 mL of supernatant and incubated at 40°C for 20 min. Absorbance was measured at 510 nm in a spectrophotometer, with the percent amylose and amylopectin measured as follows:

$$\begin{array}{l} \text{ Amylose }(\%)=\;\frac{{\text{Abs}}_{\left(\text{Con A supernatant}\right)}\;}{{\text{Abs}}_{\left(\text{Total starch aliquot}\right)}\;}\times \frac{6.15}{9.2}\times 100,\\ \text{Amylopectin }(\%)=100\%-\text{Amylose }(\%), \end{array}$$

where 6.15 and 9.2 are dilution factors for the Con A and total starch extracts, respectively.

### Statistical Analysis

Lentil, common bean, and chickpea market classes and replicates were considered as random factors and included as class variables. Analysis of variance (ANOVA) was performed using the General Linear Model procedure (PROC GLM) of SAS version 9.4 (36) and Fisher's protected least significant difference (LSD) at *P* < 0.05 was used to separate means.

## Results

Total carbohydrate concentrations averaged 51/100 g in lentil, 53/100 g in common bean, and 54/100 g in chickpea, while total prebiotic carbohydrates averaged 12/100 g in lentil, 15/100 g in common bean, and 12/100 g in chickpea (Table 2). Sugar alcohols and oligosaccharide concentrations were generally higher in lentil whereas hemicellulose, cellulose, resistant starch, amylose, and amylopectin were slightly higher in common bean and chickpea.

**Table 2:** Prebiotic carbohydrate profiles of lentil, common bean, and chickpea.

**Carbohydrates**	**Lentil**	**Dry bean**	**Chickpea**
Sugar alcohols (mg/100 g)	707 ± 51	11 ± 3	548 ± 53
**Simple sugars**			
Monosaccharides (mg/100 g)	44 ± 23	66 ± 15	34 ± 4
Disaccharides (g/100 g)	1.7 ± 0.4	3.1 ± 0.4	2.2 ± 0.4
**Oligosaccharides**			
Raffinose family oligosaccharides (g/100 g)	4.1 ± 0.5	3.0 ± 0.3	2.1 ± 0.2
Fructooligosaccharides (mg/100 g)	333 ± 80	52 ± 13	46 ± 16
**Polysaccharides**			
Hemicellulose (g/100 g)	3.8 ± 0.2	7.9 ± 0.5	6.1 ± 0.5
Cellulose (g/100 g)	0.5 ± 0.2	1.6 ± 0.9	1.1 ± 0.3
Soluble starch (g/100 g)	40 ± 3	41 ± 3	42 ± 4
Resistant starch (g/100 g)	2.1 ± 0.3	2.4 ± 0.4	3.1 ± 0.1
Amylose (g/100 g)	17 ± 2	19 ± 2	19 ± 2
Amylopectin (g/100 g)	25 ± 2	24 ± 2	26 ± 2
Unidentified Prebiotic carbohydrates (mg/100 g)	426 ± 39	151 ± 28	183 ± 80
Total prebiotic carbohydrates (g/100 g)	12 ± 1	15 ± 1	12 ± 2
Total identified carbohydrates (g/100 g)	51 ± 2	53 ± 2	54 ± 7
RDA from a 100 g serving (%)	60 ± 6	75 ± 5	60 ± 8

*Data represent mean value ± standard deviation. Values are presented on a wet weight basis (10%). Recommendations for safe daily total prebiotic intake (20 g/day) reported by Douglas and Sanders (37). Unidentified compound concentrations were determined based on of those identified carbohydrate peak areas that were very closest to retention times*.

### Lentil

**Figure 1:**
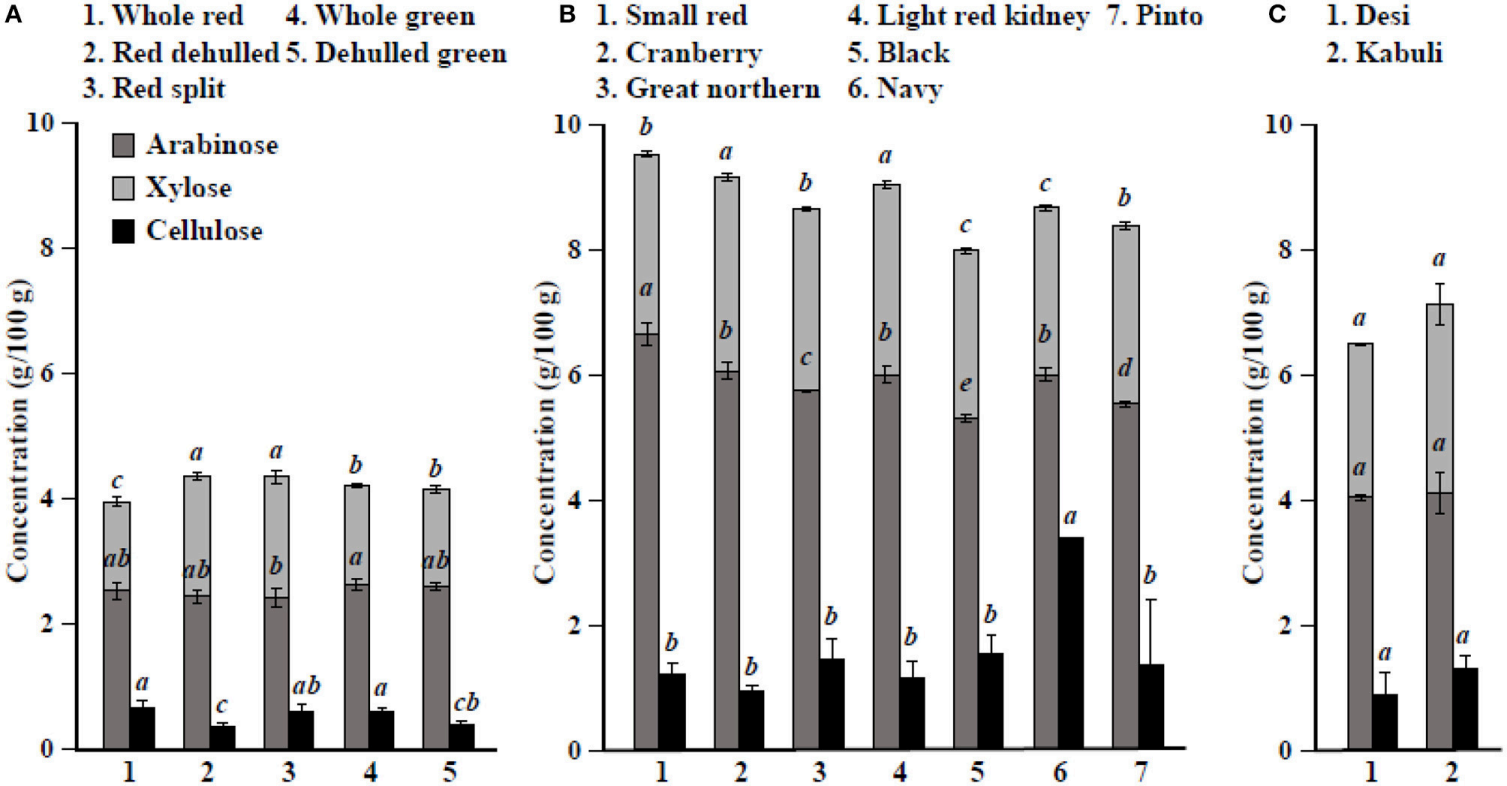
Hemicellulose (arabinose+xylose) and cellulose concentrations in different **(A)** lentil, **(B)** common bean, and **(C)** chickpea market classes. Values are presented on a wet weight basis (10% moisture). Values within each market class followed by a different letter are significantly different at *P* < 0.05 (*n* = 42).

**Figure 2:**
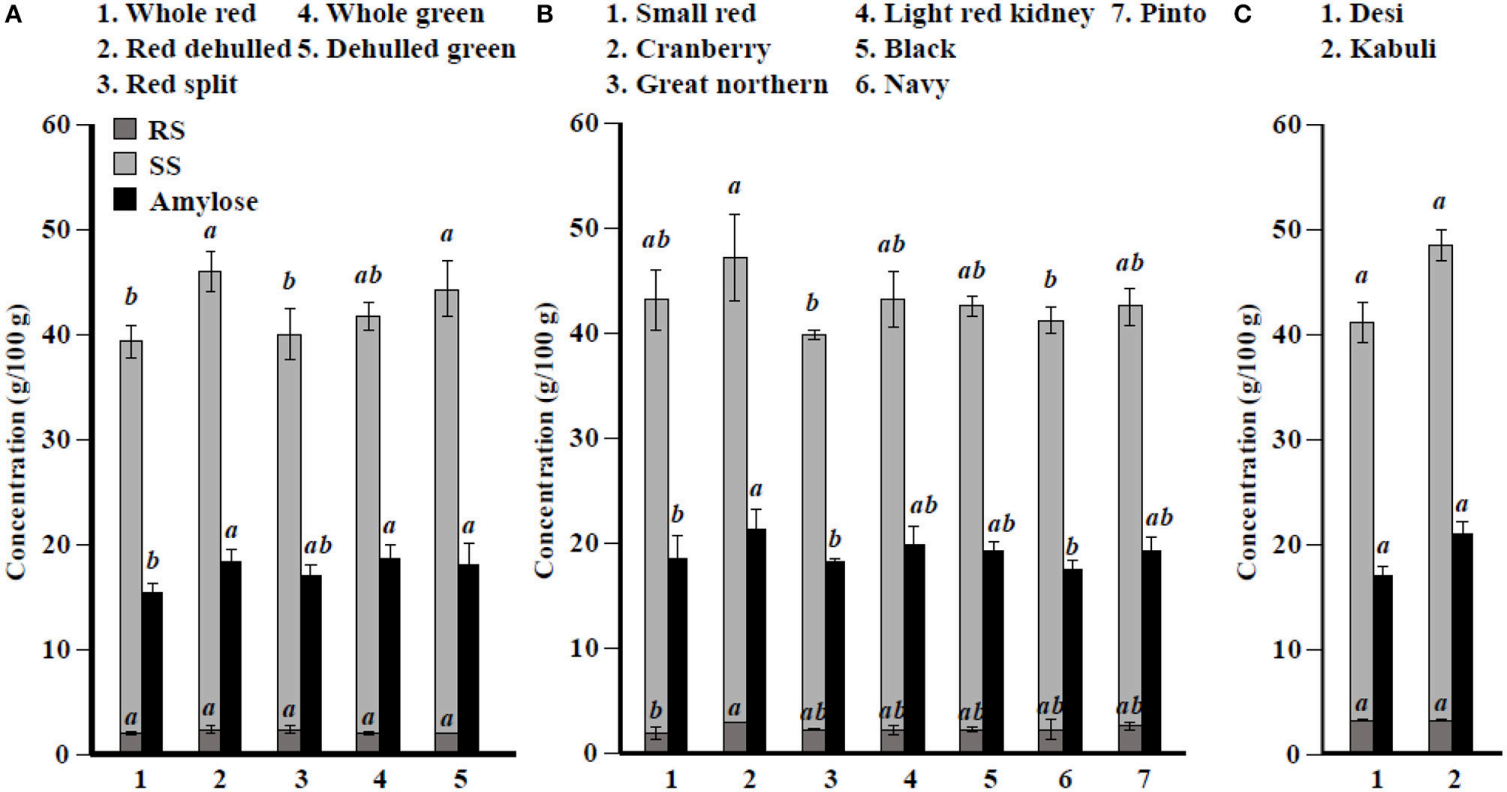
Soluble starch (SS), resistant starch (RS), and total amylose concentration in different **(A)** lentil, **(B)** common bean, and **(C)** chickpea market classes. Values are presented on wet weight basis (10% moisture). Values within each market class followed by a different letter are significantly different at *P* < 0.05 (*n* = 42).

Among simple sugars, sucrose was the most abundant (1.17–2.289/100 g) followed by glucose (21–61 mg/100 g), fructose (0.2–21.9 mg/100 g), mannose (1.2–7.9 mg/100 g), and rhamnose (0.5–1.0 mg/100 g) (Table 3). For SAs, lentil contained higher concentrations of sorbitol (606–733 mg/100 g) than mannitol (9–31 mg/100 g) and xylitol (14–31 mg/100 g) regardless of market class (Table 4). Whole red had significantly (*P* < 0.05) higher levels of sorbitol than all other market classes, and whole green had significantly higher mannitol and xylitol concentrations. For RFO, stachyose concentrations (2.24–2.35/100 g) were higher than raffinose (403–646 mg/100 g) and verbascose (581–1,769 mg/100 g) concentrations (Table 5). Considering lentil FOS, concentrations of kestose were considerably higher than those for nystose. Arabinose concentrations were significantly higher in whole green compared to red split lentil (Figure 1A). Among the market classes, red dehulled, and red split had significantly higher xylose concentrations (1,912–1,936 mg/100 g) than the other market classes. Whole red and whole green had significantly higher cellulose concentrations (611–640 mg/100 g) than the other market classes (Figure 1A). Soluble starch concentrations ranged from 37 to 44/100 g with levels in red dehulled and dehulled green significantly higher than those in whole red and red split (Figure 2A). No significant differences were observed for RS levels among market classes; however, amylose concentrations were significantly higher in red dehulled, whole green, and dehulled green than in whole red (Figure 2A).

**Table 3:** Concentration of simple sugars of different lentil, common bean, and chickpea market classes.

**Market class**	**Concentration (mg/100 g)**				
	**Mannose**	**Glucose**	**Fructose**	**Sucrose**	**Rhamnose**
**LENTIL**					
Whole red	1.5 ± 0.7c	60.5 ± 7.7a	21.9 ± 2.6a	1, 174 ± 89e	0.7 ± 0.2b
Red dehulled	5.6 ± 0.3b	24.6 ± 1.3c	0.5 ± 0.1c	2, 057 ± 94b	0.5 ± 0.0b
Red split	7.9 ± 0.9a	21.1 ± 1.0c	0.3 ± 0.1c	2, 288 ± 76a	0.7 ± 0.2b
Whole green	1.2 ± 0.3c	42.2 ± 5.4b	4.5 ± 2.0b	1, 665 ± 25c	1.0 ± 0.0a
Dehulled green	1.8 ± 0.2c	24.3 ± 4.8c	0.2 ± 0.1c	1, 376 ± 140d	0.5 ± 0.0b
Mean	3.6 ± 2.8	34.6 ± 16.0	5.5 ± 8.8	1, 712 ± 435	0.7 ± 0.2
**COMMON BEAN**					
Small red	9.5 ± 7.0a	57.9 ± 8.9ab	12.6 ± 6.6a	3, 287 ± 115b	0.2 ± 0.0c
Cranberry	3.6 ± 2.0cb	54.6 ± 6.9cb	5.4 ± 5.2cb	3, 710 ± 73a	0.7 ± 0.1a
Great northern	10.5 ± 1.0a	46.5 ± 2.9ed	5.2 ± 1.4cb	3, 296 ± 116b	0.1 ± 0.0c
Light red kidney	7.9 ± 2.6ab	49.9 ± 3.9cd	12.6 ± 8.6a	3, 188 ± 29b	0.3 ± 0.0b
Black	1.5 ± 0.1c	62.1 ± 4.1a	16.4 ± 0.9a	2, 605 ± 94c	0.2 ± 0.0c
Navy	11.2 ± 0.8a	41.8 ± 0.4ef	1.7 ± 0.7c	2, 637 ± 30c	0.2 ± 0.0c
Pinto	1.7 ± 0.6c	34.7 ± 2.4f	10.0 ± 0.9ab	2, 660 ± 113c	0.1 ± 0.0c
Mean	6.6 ± 4.7	49.6 ± 10.0	9.1 ± 6.2	3, 055 ± 412	0.3 ± 0.2
**CHICKPEA**					
Desi	0.8 ± 0.2a	29.6 ± 6.4a	2.2 ± 0.2a	1, 764 ± 104b	0.1 ± 0.0a
Kabuli	0.5 ± 0.1b	31.8 ± 0.6a	2.5 ± 0.3a	2, 541 ± 69a	0.1 ± 0.0a
Mean	0.6 ± 0.2	31.7 ± 4.2	2.4 ± 0.3	2, 153 ± 433	0.1 ± 0.0

*Data represent mean value ± standard deviation. Values are presented on a wet weight basis (10% moisture). Values within each market class followed by a different letter are significantly different at P < 0.05 (n = 42)*.

**Table 4:** Concentration of sugar alcohols (sorbitol, mannitol, and xylitol) of different lentil, common bean, and chickpea market classes.

**Market class**	**Concentration (mg/100 g)**		
	**Sorbitol**	**Mannitol**	**Xylitol**
**LENTIL**			
Whole red	733 ± 44a	9 ± 1d	14 ± 1d
Red dehulled	606 ± 24c	21 ± 1c	24 ± 1c
Red split	649 ± 23cb	22 ± 4c	28 ± 1b
Whole green	631 ± 7cb	31 ± 1a	31 ± 1a
Dehulled green	690 ± 61ab	27 ± 4b	22 ± 2c
Mean	662 ± 56	22 ± 8	24 ± 6
**COMMON BEAN**			
Small red	0.8 ± 0.0c	4.1 ± 0.3c	3.8 ± 0.1c
Cranberry	0.7 ± 0.0c	8.8 ± 0.6b	1.9 ± 0.3e
Great northern	0.2 ± 0.0e	3.7 ± 0.3cd	4.9 ± 0.3b
Light red kidney	0.1 ± 0.1e	12.7 ± 0.3a	3.1 ± 0.1d
Black	2.3 ± 0.2a	3.1 ± 0.1ed	8.6 ± 0.3a
Navy	0.4 ± 0.1d	3.0 ± 0.4e	3.5 ± 0.4cd
Pinto	1.2 ± 0.2b	3.2 ± 0.1ed	3.7 ± 0.4c
Mean	0.8 ± 0.7	5.5 ± 3.6	4.2 ± 2.0
**CHICKPEA**			
Desi	557 ± 16a	19 ± 6a	18 ± 1a
Kabuli	473 ± 8b	15 ± 5a	14 ± 0b
Mean	515 ± 48	17 ± 6	16 ± 2

*Data represent mean value ± standard deviation. Values are presented on a wet weight basis (10% moisture). Values within each market class followed by a different letter are significantly different at P < 0.05 (n = 42)*.

**Table 5:** Raffinose family oligosaccharides (raffinose, stachyose, and verbascose) and fructooligosaccharides (kestose and nystose) concentrations in different lentil, common bean, and chickpea market classes.

**Market class**	**RFO (mg/100 g)**			**FOS (mg/100 g)**	
	**Raffinose**	**Stachyose**	**Verbascose**	**Kestose**	**Nystose**
**LENTIL**					
Whole red	492 ± 119ab	2294 ± 35a	581 ± 51c	191 ± 16b	0.01 ± 0.00b
Red dehulled	464 ± 32ab	2236 ± 107a	1435 ± 74b	349 ± 20a	0.01 ± 0.00b
Red split	646 ± 144a	2348 ± 198a	1769 ± 43a	391 ± 25a	0.01 ± 0.00b
Whole green	477 ± 26ab	2290 ± 71a	1653 ± 68a	382 ± 2a	0.01 ± 0.00b
Dehulled green	403 ± 96b	2292 ± 66a	1333 ± 153b	353 ± 61a	0.08 ± 0.04a
Mean	496 ± 116	2292 ± 100	1354 ± 437	333 ± 80	0.02 ± 0.03
**COMMON BEAN**					
Small red	721 ± 114a	2492 ± 62a	128 ± 31b	45 ± 4cb	0.01 ± 0.00a
Cranberry	644 ± 65ab	2436 ± 70ab	187 ± 16a	56 ± 4ab	0.01 ± 0.00a
Great northern	626 ± 47ab	2315 ± 8b	157 ± 21ab	43 ± 6cb	0.01 ± 0.00a
Light red kidney	717 ± 31a	2093 ± 30c	181 ± 16a	69 ± 4a	0.01 ± 0.00a
Black	754 ± 103a	2404 ± 130ab	166 ± 24ab	68 ± 14a	0.01 ± 0.00a
Navy	642 ± 31ab	2011 ± 71c	187 ± 31a	45 ± 9cb	0.01 ± 0.00a
Pinto	532 ± 52b	1774 ± 41d	171 ± 15ab	38 ± 1c	0.01 ± 0.00a
Mean	662 ± 93	2218 ± 258	168 ± 28	52 ± 13	0.01 ± 0.00
**CHICKPEA**					
Desi	340 ± 51b	1437 ± 58b	113 ± 24a	55 ± 10a	2 ± 2a
Kabuli	543 ± 48a	1629 ± 6a	127 ± 39a	25 ± 6a	9 ± 6a
Mean	441 ± 120	1533 ± 112	120 ± 30	40 ± 18	5 ± 6

*Data represent mean value ± standard deviation. Values are presented on a wet weight basis (10% moisture). Values within each market class followed by a different letter are significantly different at P < 0.05 (n = 42)*.

### Common Bean

Among simple sugars, sucrose was the most abundant (2,605–3,710 mg/100 g) followed by glucose (35–62 mg/100 g), fructose (1.7–16.4 mg/100 g), mannose (1.5–11.2 mg/100 g), and rhamnose (0.1–0.7 mg/100 g) (Table 3). Considering SAs, common beans had higher concentrations of mannitol (3–13 mg/100 g) than sorbitol (0.1–2.3 mg/100 g) and xylitol (1.9–8.6 mg/100 g) (Table 4). Among market classes, light red kidney bean had significantly (*P* < 0.05) higher mannitol concentrations and black bean had higher sorbitol and xylitol concentrations. Considering common bean RFO, stachyose concentrations were higher (1.77–2.49/100 g) than those for raffinose (532–754 mg/100 g) and verbascose (128–187 mg/100 g) (Table 5). For FOS, kestose concentrations (38–69 mg/100 g) were higher than nystose concentrations (0.01–0.01 mg/100 g) (Table 5). Common bean arabinose and xylose concentrations ranged from 5.3 to 6.6/100 g and 2.7–3.1/100 g, respectively (Figure 1B). Among common bean market classes, small red had significantly more (*P* < 0.05) arabinose and cranberry bean and light red kidney bean had significantly more (*P* < 0.05) xylose. Cellulose concentrations ranged from 0.9 to 3.4/100 g, with navy bean having the highest concentration (Figure 1B). Soluble starch, RS, and amylose concentrations ranged from 38 to 44, 2 to 3, and 18 to 21/100, respectively. Overall, cranberry bean had higher SS, RS, and amylose concentrations (Figure 2B).

### Chickpea

Sucrose was the most abundant simple sugar (1.76–2.54/100 g) in chickpea, followed by glucose (30–32 mg/100 g), fructose (2.2–2.5 mg/100 g), mannose (0.5–0.8 mg/100 g), and rhamnose (0.1–0.1 mg/100 g) (Table 3). Among chickpea SAs, sorbitol concentrations (473–557 mg/100 g) were higher than mannitol (15–19 mg/100 g) and xylitol (14–18 mg/100 g) concentrations (Table 4). Overall, desi had higher sorbitol, mannitol, and xylitol concentrations than kabuli; however, differences were only significant for sorbitol and xylitol (*P* < 0.05). Among RFO in chickpea, stachyose concentrations (1.44–1.63/100 g) were higher than raffinose (340–543 mg/100 g) and verbascose (127–113 mg/100 g) concentrations (Table 5). Kabuli had significantly more (*P* < 0.05) raffinose and stachyose than desi. Considering FOS in chickpea, kestose concentration (25–55 mg/100 g) was higher than nystose concentration (2–9 mg/100 g) (Table 5). Arabinose, xylose, cellulose, SS, RS, and amylose concentrations ranged from 4.0 to 4.1, 2.5 to 3.0, 0.9 to 1.3, 38 to 45, 3.1 to 3.1, and 17 to 21/100 g, respectively, but none of these were significantly different between desi and kabuli (Figures 1C, 2C).

## Discussion

Pulses, including lentil, common bean, and chickpea, are traditional staple foods that have been consumed for several centuries because of their superior nutritional profile (9, 38–40). However, increasing global demand for highly processed sugar and fat-rich foods has led to severe non-communicable disease epidemics, including obesity, overweight, and cancer (41). A diet rich in prebiotic carbohydrates, low in energy and glycemic response, moderate in protein, low in fat, and rich in micronutrients is now recommended for weight management (42). Cereal-based diets can satisfy daily caloric requirements, but do not provide daily requirements of prebiotic carbohydrates in a single serving (43). The present study indicates that pulses (lentil, common bean, and chickpea) provide 60–75% of the daily safe requirement of prebiotic carbohydrates (20 g/day) in a single serving [Table 2; (37)]. The official recommendations have not been made yet for prebiotic carbohydrate consumption, however several researches have offered suggestions for safe intake (37). Additionally, this current work provides information on the types and quantities of prebiotic carbohydrates in—different pulse market classes, which is valuable for further enhancement of nutritional via plant breeding and genetic selection.

Simple sugar concentrations in lentil, common bean, and chickpea are comparable to previous studies (44). Simple sugar concentrations in common bean were higher than in lentil and chickpea. In contrast, SA concentrations were higher in lentil and chickpea than in common bean. Simple sugars are precursors of SA formation in plants; however, this negative correlation between simple sugars and SA is largely dependent on plant type and weather conditions (45). Simply, from 5.1 to 6.7, 1.7 to 2.6, and 2.1 to 2.8/100 g for RFO (46–48) and 0.0 to 0.7, 0.0 to 0.5, and 0.0 to 0.07/100 g for FOS (49, 50) in lentil, common bean, and chickpea, respectively; these values are comparable to those from the current study. Further, the present study found total polysaccharides are higher in common bean and chickpea than in lentil, similar to previous reports (51, 52). The composition of carbohydrates depends on their localization in the seed coat or cotyledon (8). Cell walls of the cotyledon contain a range of polysaccharides including cellulose, starch, and non-starchy non-cellulosic glucans, while the seed coat contains large quantities of low molecular weight carbohydrates and cellulose but is low in hemicellulose (8). Lentil seeds are generally smaller than common bean and chickpea (Table 1); this might explain why increased levels of low molecular weight carbohydrates (SA, RFO, and FOS) are found in lentil while common bean and chickpea contain higher levels of cellulose and hemicellulose (Table 2).

Sucrose is the most abundant simple sugar found in pulses. During the development of the endosperm in the seed, the concentration of hexose declines while sucrose increases (53). Among lentil market classes, red lentil has higher levels of simple sugars than green lentil. Also, whole green lentil (lentil with seed coat) contains more sucrose, glucose, and fructose than dehulled green lentil, in accordance with earlier studies (40, 54) the opposite is true with respect to mannose (Table 3). In common bean, cranberry, small red, and great northern bean had higher total simple sugars while black and navy bean had the least (Table 3), showing significant variation among market classes due to structural (i.e., seed size), genetic, and environmental variations (48). Among chickpea market classes, kabuli had significantly more sucrose than desi due to its larger cotyledon size (55).

With respect to SAs, whole red lentil had higher sorbitol than dehulled lentil and dehulled red lentil had higher mannitol and xylitol; however, the opposite is true for green lentil, showing that SA distribution in lentil seed is influenced by both market class (red vs. green) and processing method (whole vs. dehulled), as noted previously (56). Common bean market classes also varied with respect to SA levels and had more mannitol and xylitol than sorbitol. Light red kidney bean, which has the largest seed size among studied market classes, had 50% more SA than all other market classes. In chickpea, desi (smaller seed size, and hence more seed coat area) had more SA than kabuli, which is attributed to the more SA being present in seed coat than the cotyledon. Across all three pulse crop types, SA varied with seed size, market class, and processing method.

Lentil RFO concentration varies with genotype and growing environment (9, 50). Moreover, dehulling generally reduces raffinose concentrations but increases stachyose and verbascose concentrations (40, 47, 56). In the current study, dehulling only increased verbascose concentration in red lentil. The greater variation in stachyose vs. raffinose and verbascose levels among common bean market classes might be due to genetic differences. Along with variations in seed size, seed coat thickness, and surface area, genetic makeup might affect the RFO concentration in common bean. In chickpea, more RFOs were found in kabuli (55), which has a large seed size and hence a larger seed cotyledon (Tables 1, 5). With respect to FOS, our data show higher levels of kestose present in the seed cotyledon than the seed coat in red lentil, with the reverse observed in green lentil (Table 5). Kestose levels varied significantly among common bean and chickpea market classes, indicating that kestose synthesis might be influenced by market class (57).

The seed coat contains most of the cellulose found in the seed (58). Our data confirm that whole lentil generally had higher cellulose levels than dehulled lentil. Similarly, arabinose and xylose were slightly higher in whole lentil and dehulled lentil, respectively, reflecting differences in the distribution of hemicellulose compounds in the seed. Cellulose levels are higher in common bean market classes when the seed size decreases, suggesting cellulose compounds are abundant in the seed coat. In contrast, arabinose and xylose levels are positively correlated with seed size. In chickpea, significant differences between desi and kabuli were not observed, which contrasts with previously reported results (52). Lentil dehulling slightly increases RS and SS as dehulling removes the starch-free seed coat, therefore concentrating starch fractions in the seed cotyledon (47, 56). In common bean and chickpea market classes, RS and SS are positively correlated with seed size (Figure 2B, Table 1), which relates to where starch compounds are stored in the cotyledon. Further, data from the current study confirm the positive correlation of amylose concentrations with RS, SS, and total starch (sum of RS and SS), similar to previous reports (59). Johnson et al. (47) indicated that significant changes in lentil RS concentration due to processing, cooking, and cooling. Cooling of cooked lentil increased RS concentration approximately two-fold from 3.0% (w/w) in cooked lentil to 5.5% (w/w) after cooling. Further, RS concentrations ranged from 3 to 5% (w/w) in raw lentil and the concentrations of RS in raw and cooked lentils were not significantly different (47). This current study reports only dry pulse seed RS concentrations for future breeding and selection purposes.

Overall, prebiotic carbohydrates represented 24, 28, and 22% of the total carbohydrate compounds in lentil, common bean, and chickpea, respectively. Prebiotic carbohydrate concentrations differ among pulses due to seed size, type of pulse, and processing method, and therefore incorporation of several pulses in the diet provides a range of different prebiotic carbohydrates needed for gut health. However, this present study did not report several prebiotic carbohydrates including pectin, and types of hemicellulose which does occur in most legume seeds. Further, complete profiling of carbohydrates in pulses provides useful information for future plant breeding and genetic studies to understand the prebiotic carbohydrate control mechanism in plants (60).

## Conclusion

This study shows the type and quantity of prebiotic carbohydrates varies with pulse crop, market class, seed size, and processing method. Lentil, common bean, and chickpea provide 60–75% of the suggested daily intake of prebiotic carbohydrates in a 100 g serving. Lentil is rich in low molecular weight carbohydrates including SA, RFO, and FOS, while common bean and chickpea are rich in polysaccharides such as cellulose, hemicellulose, and amylose. Overall, these pulses are rich in prebiotic carbohydrates, and further nutritional breeding is possible with identifying suitable growing locations, and genotypes producing higher levels of prebiotic carbohydrates in different pulse crop market classes.

## Author Contributions

NS: graduate student worked on the project - created all data, data analysis, and prepared first draft of this manuscript; PT: developed analytical assays, data analysis; SK: experimental design, manuscript editing; DT: PI of the project, graduate student supervision, data analysis, data interpretation, and manuscript writing and editing final version.

### Conflict of Interest Statement

The authors declare that the research was conducted in the absence of any commercial or financial relationships that could be construed as a potential conflict of interest.

## Abbreviations

Abs: Absorbance
ANOVA: Analysis of variance
C_f_: Concentration of filtrate
C_s_: Concentration of sample
ddH_2_O: Distilled and deionized water
FOS: Fructooligosaccharides
HPAE: High performance anion exchange chromatography
LMWC: Low molecular weight carbohydrates
LSD: Least significant difference
m: Mass of the sample
PAD: Pulsed amperometric detector
RDA: Recommended daily allowance
RFO: Raffinose family oligosaccharides
RS: Resistant starch
SA: Sugar alcohol
SS: Soluble starch
V: Volume of sample.
